# Pseudo-Meigs syndrome secondary to endodermal sinus tumor

**DOI:** 10.14744/nci.2020.45452

**Published:** 2022-12-15

**Authors:** Dilan Altintas Ural, Ali Erdal Karakaya, Ahmet Gokhan Guler, Can Acipayam, Mustafa Sabih Kaya, Mehmet Cihan Karacaoglu, Sezen Kocarslan

**Affiliations:** 1 Department of Pediatric Surgery, Kahramanmaras Sutcu Imam University Faculty of Medicine, Kahramanmaras, Turkey; 2 Department of Child Health and Diseases, Kahramanmaras Sutcu Imam University Faculty of Medicine, Kahramanmaras, Turkey; 3 Department of Radiology, Kahramanmaras Sutcu Imam University Faculty of Medicine, Kahramanmaras, Turkey; 4 Department of Pathology, Kahramanmaras Sutcu Imam University Faculty of Medicine, Kahramanmaras, Turkey

**Keywords:** Acid, pleural effusion, pseudo-Meigs Syndrome, yolk sac tumor

## Abstract

Ovarian tumors are the most common gynecological tumors seen in girls. Approximately 60–70% of them are germ cell tumors. Pseudo-Meigs syndrome is characterized by the presence of pelvic tumoral mass (benign or malign), pleural effusion, and massive acid. If the tumor is removed, acid and hydrothorax disappear. Endodermal sinus (yolk sac) tumor is a very rare cause in the diagnosis of Pseudo-Meigs syndrome, and only a few cases have been reported. This case is one of the rare cases presenting with Pseudo-Meigs syndrome and pathologically diagnosed as yolk sac tumor.

**T**he neoplastic masses originated from ovaries in the childhood constitute almost half of all ovarian tumors. Almost 64% of these masses are neoplastic and 10–40% are malignant [1]. Childhood ovarian masses are most frequently cystic and may be of solid and mixed types. Most of the masses are placed on the right ovary. Meigs syndrome is caused by benign ovarian mass such as typical fibroma, granulosa cell tumor, thecoma, or Brenner tumor with acid and pleural effusion. Pseudo Meigs syndrome is characterized by a pelvic mass (benign or malign), pleural effusion, and massive acid [2, 3]. Endodermal sinus tumor (yolk sac tumor) is a very rare cause in the diagnosis of Pseudo-Meigs syndrome [4]. This case is one of the rare cases of pseudo-Meigs syndrome with pathological and radiological intervention.

## Case Report

**Figure 1.**
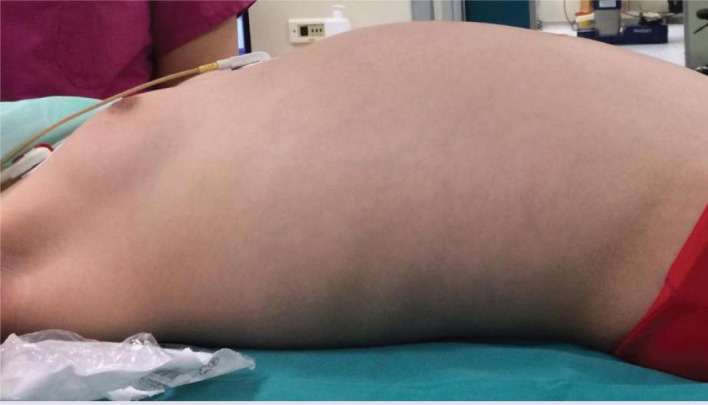
The patient had significant abdominal distention on physical examination.

A 12-year-old girl was brought with abdominal pain, which started about 10 days ago, with a complaint of abdominal mass. The patient had significant abdominal distention on physical examination (Fig. 1). In diagnosis, abdominal ultrasonography (USG) was performed first. Abdominal computed tomography (CT) and abdominal magnetic resonance imaging (MRI) were performed as advanced imaging methods. On chest X-ray, an effusion was detected in the right hemithorax.

**Figure 2.**
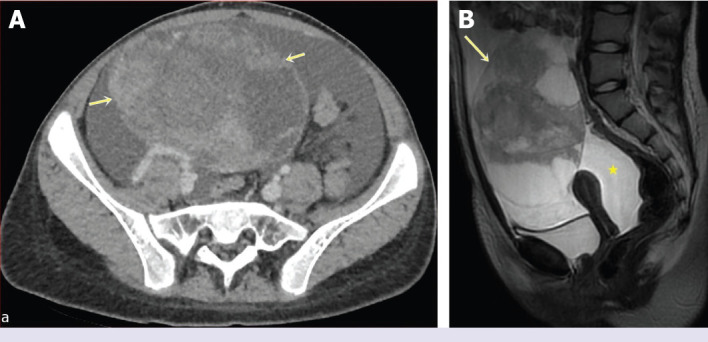
**(A)** Contrasted computed tomography shows the heterogeneous mass (arrow) originating from the right ovary. **(B)** Sagittal T2- magnetic resonance image, irregular soft tissue components show significant contrast increase (arrow), accompanying diffuse intraperitoneal free fluid is also seen (star), no abnormality in uterus.

Abdominal USG determined a 17 × 10 cm vascular solid lesion in the pelvis and widespread fluid in the pelvis at a depth of 11 cm. In contrast-enhanced whole abdominal CT, a cystic necrotic lesion with a solid component starting from the right part of the pelvis and contrasting multiple irregularly at a size of 17 × 13 × 9 cm, and 10 cm free acid in the pelvis was detected (Fig. 2a). MR detected a massive mass lesion of 15 × 12 × 9 cm extending in the inferior bladder and uterus in the abdomen, 10 cm free fluid in the pelvis (Fig. 2b). The patient’s tumor markers were as follows: beta subunit of human chorionic gonadotropin (β-HCG): 4.3 mlu/ml [2–6], alpha fetoprotein (AFP): 26900.0 IU/ml (0–5.8).

**Figure 3.**
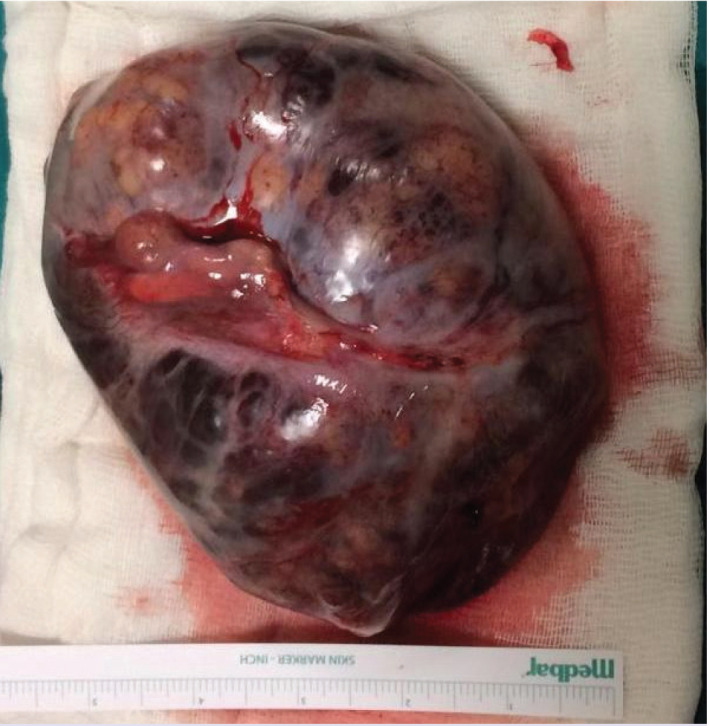
Ovarian tumor with macroscopic smooth lobed surface, gray-yellow, necrotic and hemorrhagic areas was completely removed.

**Figure 4.**
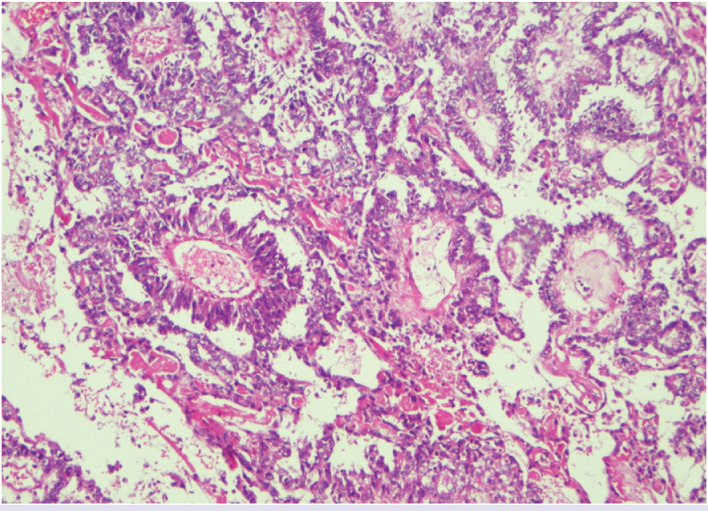
In the histopathological section of the ovarian mass, the Yolk Sak tumor consisting of tubular capsular sinusoidal structures (Schiller-Duval body) containing central fibrovascular core covered with cuboidal-columnar cells (Hematoxylin and Eosin 100×).

Informed consent was obtained from the patient’s parents. Following approval of informed consent and operation preparations, the patient underwent surgery. Approximately 4.5 l of free acid was aspirated in the operation. The mass adhering to the liver originating from the right ovary and adhering to the omentum from the upper part with a smooth contour was removed by right salpingo-oophorectomy (Fig. 3). Samples were taken from the peritoneum and omentum. The pathological diagnose was reported as a yolk sac tumor with a size of 15 × 13 × 8 cm (Fig. 4). Omentum and peritoneal tissues were biopsied. In fluid histopathology, mesothelial cells and rare lymphocytes were seen. The patient was given Bleomycin- Etoposide- Cisplatin (BEP) chemotherapy protocol by pediatric oncology.

## Discussion

Ovarian tumors are the most common gynecological tumors in children and are approximately 1% of all childhood cancers. About 40–50% of the ovarian masses are neoplastic, and 60–70% of these are germ cell tumors. Epithelial tumors are seen rarely (10–15%). Germ cell tumors develop from embryonic gonad primitive germ cells. These tumors include dysgerminoma, yolk sac tumor, embryonal carcinoma, mature teratoma, immature teratoma, choriocarcinoma, and malignant mixed germ cell tumors. All germ cell tumors are malignant except mature teratoma [5].

Endodermal sinus tumor (yolk sac tumor) is a very rare cause in the diagnosis of Pseudo-Meigs syndrome, and only a few cases have been reported [6, 7]. The cause of the acid has not been cleared, it is thought to be due to the overtransudation than its absorption capacity towards the tumor surface. Another reason is the formation of a peritonitis due to the growth of the tumor [8].

The most common symptoms of ovarian tumors are abdominal pain and mass. Other findings are urinary accumulation, constipation, anorexia, vomiting, and intestinal obstruction due to the compression effect of the mass. It has aggressive growth and spreading potential. Tumor size can be up to 30 cm in diameter, with an average diameter of 15.5 cm. In most patients, symptoms appear in less than a week. If complications such as torsion, cyst rupture, and perforation occur, the patient suffers from acute abdominal syndrome [5].

USG gives information about the size, structure, and localization of the ovary. Advanced imaging methods (CT and MRI) are recommended to assess pelvic anatomy, paraaortic lymph nodes, and detect the presence of metastases [9].

Tumor markers are important in postoperative follow-up and regression of the disease. AFP, β-HCG, cancer antigen (CA)-125, lactate dehydrogenase, carcinoembryogenic antigen and CA-19-9 levels are less specific in children than in adults for the ovarian malign tumors. In contrast, beta HCG and AFP are more sensitive in children. Yolk sac tumor of ovary releases AFP. In our case, the level of AFP was quite high (AFP: 26900.0 IU/ml (0–5.8).

Yolk sac tumor may be associated with other germ cell tumors. Microscopically the most common subtype is the reticular type which has a structure named Schiller-Wall body. This structure consists of primitive cells around a capillary. The treatment requires surgery. In fertility-sparing surgery, unilateral oophorectomy, salpingo-oophorectomy and intraabdominal tumor debulking are the possible techniques to remove the gross tumors. BEP and Vincristine- Actinomycin D- Cyclophosphamide are commonly used chemotherapy regimens [10].

### Conclusion

Although rarely seen in the causes of Pseudo-Meigs syndrome in girls, yolk sac tumor should also be considered in the differential diagnosis. Fertility-sparing surgery should be planned as the most appropriate treatment and patients should be followed up for long-term.
